# Differential Cytokine Responses in Hospitalized COVID-19 Patients Limit Efficacy of Remdesivir

**DOI:** 10.3389/fimmu.2021.680188

**Published:** 2021-06-28

**Authors:** Yi-Hao Chan, Barnaby E. Young, Siew-Wai Fong, Ying Ding, Yun Shan Goh, Rhonda Sin-Ling Chee, Seow-Yen Tan, Shirin Kalimuddin, Paul A. Tambyah, Yee-Sin Leo, Lisa F. P. Ng, David Chien Lye, Laurent Renia

**Affiliations:** ^1^ A*STAR Infectious Diseases Labs (A*STAR ID Labs), Agency for Science, Technology and Research, Singapore, Singapore; ^2^ Singapore Immunology Network, Agency for Science, Technology and Research, Singapore, Singapore; ^3^ National Centre for Infectious Diseases, Singapore, Singapore; ^4^ Department of Infectious Diseases, Tan Tock Seng Hospital, Singapore, Singapore; ^5^ Lee Kong Chian School of Medicine, Nanyang Technological University, Singapore, Singapore; ^6^ Department of Infectious Diseases, Changi General Hospital, Singapore, Singapore; ^7^ Department of Infectious Diseases, Singapore General Hospital, Singapore, Singapore; ^8^ Emerging Infectious Diseases Program, Duke-NUS Medical School, Singapore, Singapore; ^9^ Department of Medicine, National University Hospital, Singapore, Singapore; ^10^ Saw Swee Hock School of Public Health, National University of Singapore and National University Health System, Singapore, Singapore; ^11^ Yong Loo Lin School of Medicine, National University of Singapore and National University Health System, Singapore, Singapore; ^12^ Department of Biochemistry, Yong Loo Lin School of Medicine, National University of Singapore, Singapore, Singapore; ^13^ Institute of Infection, Veterinary and Ecological Sciences, University of Liverpool, Liverpool, United Kingdom

**Keywords:** COVID-19, remdesivir (GS-5734), T-cells, tissue repair, disease progression

## Abstract

A significant proportion of COVID-19 patients will progress to critical illness requiring invasive mechanical ventilation. This accentuates the need for a therapy that can reduce the severity of COVID-19. Clinical trials have shown the effectiveness of remdesivir in shortening recovery time and decreasing progression to respiratory failure and mechanical ventilation. However, some studies have highlighted its lack of efficacy in patients on high-flow oxygen and mechanical ventilation. This study uncovers some underlying immune response differences between responders and non-responders to remdesivir treatment. Immunological analyses revealed an upregulation of tissue repair factors BDNF, PDGF-BB and PIGF-1, as well as an increase in ratio of Th2-associated cytokine IL-4 to Th1-associated cytokine IFN-γ. Serological profiling of IgG subclasses corroborated this observation, with significantly higher magnitude of increase in Th2-associated IgG2 and IgG4 responses. These findings help to identify the mechanisms of immune regulation accompanying successful remdesivir treatment in severe COVID-19 patients.

## Introduction

The coronavirus disease 2019 (COVID-19) pandemic, caused by severe acute respiratory syndrome coronavirus 2 (SARS-CoV-2), has affected more than 163 million people, with death counts over 3.3 million worldwide (https://www.who.int/emergencies/diseases/novel-coronavirus-2019). Although most of the patients are either asymptomatic or have mild disease, approximately 20% of COVID-19 patients suffer from severe clinical illness, with 5% progressing to critical stage with acute respiratory distress syndrome and the need for mechanical ventilatory support (1–3). The demand on ventilators and intensive care unit (ICU) resources has overwhelmed the public health system globally, and led to an increase in fatality rates in some regions, exemplified in the Eastern Mediterranean, Southern Asia and South-East Asia regions (4). In order to circumvent the shortage of medical resources and reduce mortality, developing or re-purposing existing therapeutic strategies to ameliorate disease severity is paramount.

Although several drugs have been evaluated in late stage clinical trials, remdesivir (RDV) is the only antiviral agent to date to show efficacy in COVID-19 patients, and to be approved by the U.S. Food and Drug Administration (FDA) to treat COVID-19 (5–8). A phase 3 trial showed faster recovery time, shorter time on oxygen, and lower rates of progression to non-invasive and invasive mechanical ventilation but no mortality difference (5). However, some patients requiring low-flow oxygen continue to worsen despite RDV treatment, while RDV was not clearly beneficial for patients who require mechanical ventilation (5). We investigated the underlying cytokine responses of RDV-treated severe COVID-19 patients and characterized the differential responses associated with clinical outcomes following RDV treatment. Findings indicated the differences in T-helper cell polarization between non-intubated patients requiring oxygen and intubated patients, which help delineate the therapeutic effectiveness of RDV in a subset of severe COVID-19 patients.

## Methods

### Study Approval

All patients with COVID-19 infection treated in public hospitals were approached to join a prospective observational cohort study (the ‘PROTECT’ study). Study protocols were approved by ethics committees of the National Healthcare Group (Ref: 2012/00917) and SingHealth Centralized Institutional Review Board (Ref: 2018/3045). From this cohort, patients who received remdesivir as treatment for COVID-19 as part of clinical trials and patients with similar severity of illness were analyzed. Healthy donor samples were collected as part of an ageing study under study numbers 2017/2806 and NUS IRB 04-140 (**Supplementary Table 1**). Participants are identified by identification numbers in this study.

### Clinical Data and Biological Collection

Electronic medical records of enrolled patients were reviewed and data entered onto a standardized collection form adapted from the International Severe Acute Respiratory and Emerging Infection Consortium (ISARIC) case record form (9). Serial blood and respiratory samples were collected during hospitalization and follow-up post-discharge (days 1, 3, 7, 14, 21, and 28 after enrolment).

### Clinical Management

All patients with COVID-19 were isolated with airborne transmission precautions regardless of disease severity. Supportive therapy including supplemental oxygen and symptomatic treatment were administered as required to all remdesivir-treated patients and controls in this study. All patients in this study experienced moderate to severe hypoxia (defined as requiring fraction of inspired oxygen [FiO_2_] ≥40%) and were transferred to the intensive care unit (ICU) for high-flow oxygen *via* nasal cannula or invasive mechanical ventilation if required. Deisolation was contingent on prevalent policies from Ministry of Health, Singapore, which required resolved symptoms and two consecutive nasopharyngeal swabs >24 hours apart that were negative for SARS-CoV-2 by polymerase chain reaction (PCR) at the point of study.

### Clinical Definitions

“Responders” were defined as RDV-treated patients who did not progress to requiring mechanical ventilation, while RDV-treated patients whose condition deteriorated and required mechanical ventilation were classified as “non-responders”. Control patients were defined as patients not treated with RDV in the “PROTECT” study cohort who had similar clinical progression as RDV-treated patients and had matching days post-illness onset (PIO) for timepoint 1 and timepoint 2. All COVID-19 patients required low-flow oxygen at the start of the study.

### Multiplex Microbead-Based Immunoassay

Levels of specific immune mediators in the first plasma samples collected from severe COVID-19 patients who required supplemental oxygen before (Timepoint 1) and one week after RDV treatment (Timepoint 2) were quantified by multiplex microbead-based immunoassays. Immune mediator concentrations of control patient plasma from matching timepoint 1 (MTP1) and MTP2 were also quantified. Plasma samples were treated with 1% Triton™ X-100 solvent-detergent (SD) mix (ThermoFisher Scientific, Waltham, MA, USA) for virus inactivation. Immune mediator concentrations were determined with the Luminex™ assay, using the Cytokine/Chemokine/Growth Factor 45-plex Human ProcartaPlex™ Panel 1 (ThermoFisher Scientific). Standards and plasma from COVID-19 patients and healthy controls were incubated with fluorescent-coded magnetic beads pre-coated with respective capture antibodies in a 96-well black clear-bottom plate. After washing, biotinylated detection antibodies were incubated with the cytokine-bound beads for one hour. Finally, streptavidin-PE was added and incubated for another 30 minutes. Measurements were acquired on the FLEXMAP^®^ 3D (ThermoFisher Scientific) using xPONENT^®^ 4.0 (Luminex Corporation, Austin, TX, USA) acquisition software. Data analyses were performed on Bio-Plex Manager™ 6.1.1 (Bio-Rad Laboratories, Hercules, CA, USA). Standard curves were generated with a 5-PL (5-parameter logistic) algorithm, reporting values for both median fluorescence intensity (MFI) and concentration data.

### Anti-SARS-CoV-2 Spike Protein Specific IgG Isotyping

Detection of IgG subclasses specific against the full-length SARS-CoV-2 spike protein was performed using fluorescence-activated cell sorting (FACS) based assay (10). Cells expressing full-length SARS-CoV-2 spike protein were seeded at 1.5 x 10^5^ cells per well in 96 well plates (ThermoFisher Scientific). The cells were first incubated with plasma samples from COVID-19 patients and healthy controls (1:100 dilution in 10% fetal bovine serum, FBS), followed by a secondary incubation with a double stain, consisting of Alexa Fluor 647-conjugated anti-human IgG1, IgG2, IgG3 or IgG4 (ThermoFisher Scientific; 1:500 dilution in 10% FBS) and propidium iodide (Sigma-Aldrich, St. Louis, MO, USA; 1:2500 dilution). Cells were acquired on a LSRII 4 Laser flow cytometer (BD, Franklin Lakes, NJ, USA) and analyzed using FlowJo (BD). A positive antibody response cutoff is defined as mean + 3SD of the healthy controls (n=22). The ratio of the IgG subclasses was calculated with the following formula, where the percentage binding refers to the proportion of each IgG subclass that is specific to the SARS-COV-2 spike protein.

$$\text{Ratio }(\text{Ig}{\text{G}}_{2/4}/\text{Ig}{\text{G}}_{1/3})=\frac{\left(\%\text{ Binding IgG}2+\%\;\mathrm{IgG}4\right)}{\left(\%\text{ Binding IgG}1+\%\text{ IgG}3\right)}$$

### Data Processing and Statistical Analysis

Internal controls were included in each Luminex Assay to remove potential plate effects. A correction factor was obtained from the differences observed on the readouts of these samples across the multiple assays. This correction factor was then used to normalize all the samples. Cytokine concentrations out of measurement range were assigned the value of Limit of Quantification (LOQ). TM4-MeV Suite (version 10.2) was used to compute hierarchical clustering and generate a heatmap of immune mediators, scaling concentrations to between 0 and 1 for visualization. Plots were generated using GraphPad Prism version 8.2.1 (GraphPad, San Diego, CA, USA). Statistical analyses were performed with Wilcoxon matched-pairs signed rank test, Kruskal-Wallis test with Dunn’s multiple comparisons test or Mann Whitney U test as indicated. *P*-values less than 0.05 were considered to be statistically significant.

## Results

### Clinical Parameters and Effect of Remdesivir Treatment

As this was a retrospective study to elucidate the differences in immunological impact between responders and non-responders to remdesivir (RDV) treatment, samples of RDV-treated and non-RDV treated control patients were selected for comparison based on matching timepoint (days PIO) of blood sample collection and sample availability. As such, we only managed to recruit 28 patients who were treated with RDV during the course of COVID-19 in this non-randomized study. Notably, differences in gender ratio and ethnicity exists between the RDV-treated and non-RDV treated patients (**Table 1**). However, the majority of patients in both groups belonged to the Asian ethnicity. Out of these patients, 27 (96%) were receiving low-flow supplemental oxygen before the start of remdesivir treatment, with 6 progressing to invasive mechanical ventilation, or intubation (hereinafter non-responders). The remaining 21 patients remained on low-flow supplemental oxygen until eventual recovery (hereinafter responders). RDV treatment in 1 patient was initiated after intubation and excluded from subsequent analyses to reduce confounding variables. Treatment started at median 8 days (interquartile range [IQR] 6 – 10) PIO. All patients received a ≥5 day course of RDV treatment (median 10 days, IQR 10 – 10). After the initiation of RDV, 6 out of 27 patients (22%) progressed to invasive mechanical ventilation, with death occurring in 2 patients (7%). Control infected patients, who were not treated with RDV due to limited supply of doses, with matching days PIO for timepoint 1 and timepoint 2 of the treated patients were included in this study. In total, 27 control COVID-19 patients, who were receiving low-flow supplemental oxygen at matched timepoint 1 were included in this study, with 10 progressing to intubation (37%). One control patient succumbed to COVID-19 (4%).

**Table 1:** Demographics, Presenting Symptoms, Parameters, and Laboratory Investigations Stratified by Remdesivir treatment.

	Remdesivir		No Remdesivir	*P*-value
N	28		27	
Age, years	55 (45-65)		55 (42-65)	0.83
Sex	26 (93%)		16 (59%)	0.0043
Ethnicity				
-Chinese	12 (43%)		22 (81%)	
-South Asian	9 (32%)		1 (4%)	0.0063
-Other	7 (25%)		4 (15%)	
Charlson comorbidity index	0 (0-1)		0 (0-1)	0.87
-Diabetes	11 (39%)		8 (30%)	0.57
-Hypertension	16 (57%)		17 (63%)	0.78
Days from symptom onset to desaturation	8 (5-9)		8 (6-9)	0.93
Other experimental therapy				
-Anti-viral†	4 (14%)		18 (67%)	<0.0001
-Immunomodulator‡	4 (14%)		4 (15%)	1.00
	Responders (n=21)	Non-responders (n=6)	–	–
Doses (Interquartile range; median [days])	10 – 10; 10	7 – 10; 10	–	–
Invasive mechanical ventilation	7 (25%)		10 (37%)	0.39
-Duration (days, range)	- 6 (5-27)		- 8 (2-30)	
Total duration supplemental oxygen (days)	10.5 (8-18)		9 (5-13)	0.090
Duration hospital admission (days)	21.5 (16-27)		17 (13-21)	0.10
Progression to intubation	6 (22%)		10 (37%)	0.372
Death	2 (7%)		1 (4%)	1.00

^A^Data is presented as median and inter-quartile range or n (%) unless otherwise specified. Continuous variables compared with Mann-Whitney, categorical with Fisher’s exact or Chi square as appropriate.

^B^†Lopinavir/ritonavir in 19, hydroxychloroquine in 5; ‡Corticosteroids in 2, interferon in 5, tocilizumab in 2 (note some patients received more than one agent).

^C^Remdesivir-treated group were treated with 200mg loading dose on day 1, followed by a maintenance dose administered daily thereafter.

RDV-treated patients were hospitalized for a longer period (median 21.5 days, IQR 16 – 27) compared with control patients (median 17 days, IQR 13 – 21). Significantly higher proportion (93%) of patients treated with RDV were male, compared with 59% in control patients. A higher proportion of control patients were of Chinese (81%) compared with RDV-treated patients (43%). In addition, 67% of control patients were treated with other anti-viral experimental therapy, such as lopinavir/ritonavir (Kaletra), hydroxychloroquine, corticosteroids, subcutaneous interferon beta-1B and/or tocilizumab, compared with 14% in RDV-treated patients. Demographics, clinical characteristics and laboratory investigations of this retrospective cohort study are shown in **Table 1**.

### Differences in Levels of Growth and Tissue Repair Factors in Responders and Non-Responders of Remdesivir Treatment

**Figure 1:**
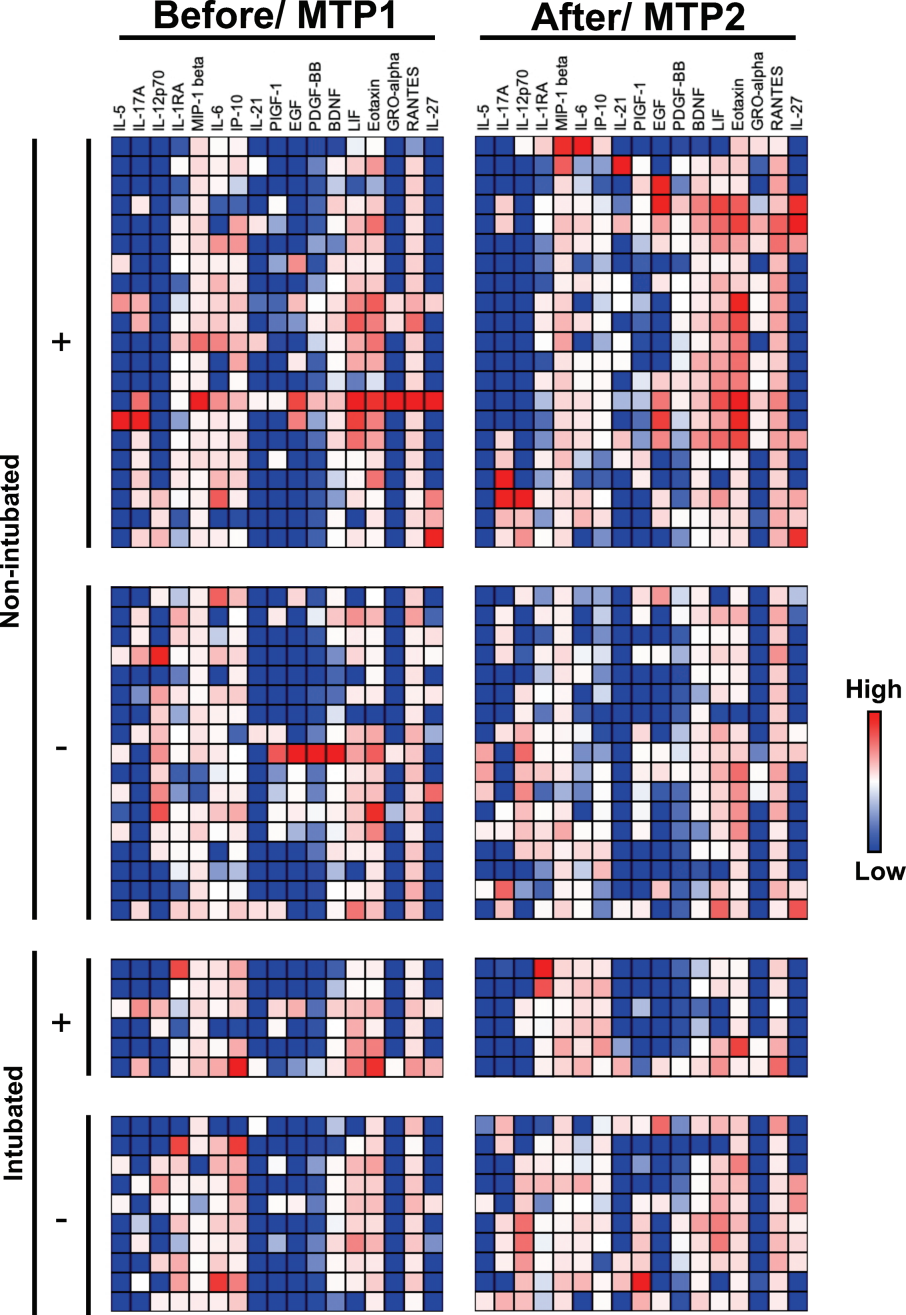
Concentrations of immune mediators in COVID-19 patients treated with remdesivir (RDV). Heatmap of immune mediator levels in plasma samples of COVID-19 patients are responders (do not progress to intubation, n = 21) or non-responders (progress to intubation, n = 6) to RDV treatment. Concentrations are measured before [median 9 and 7 days post-illness onset (PIO)] and one week (median 16 and 13 days PIO) after RDV treatment for responders and non-responders, respectively. Immune mediator levels in timepoint matched (MTP) plasmas samples from non-intubated (n = 17, median 10 and 16 days PIO) or intubated (n = 10, median 6 and 12 days PIO) COVID-19 patients were also quantified for controls. RDV-treated and control patients are indicated with + and -, respectively. Each color represents the relative concentration of a particular analyte (blue = low concentration; red = high concentration). Each row represents one patient. Patient samples with concentration out of measurement range are presented as the value of Limit of Quantification (LOQ).

To investigate the underlying cytokine responses between responders (n=21) and non-responders (n=6) of RDV treatment, immune mediator levels in plasma collected before (median 9 days and 7 days PIO, respectively) and one week after treatment (median 16 days and 13 days PIO, respectively) were profiled (**Figure 1**). In parallel, plasma immune mediators in timepoint-matched non-intubated (median 10 days and 16 days PIO) and intubated (median 6 days and 12 days PIO) non-RDV treated control patients were profiled (**Figure 1**).

**Figure 2:**
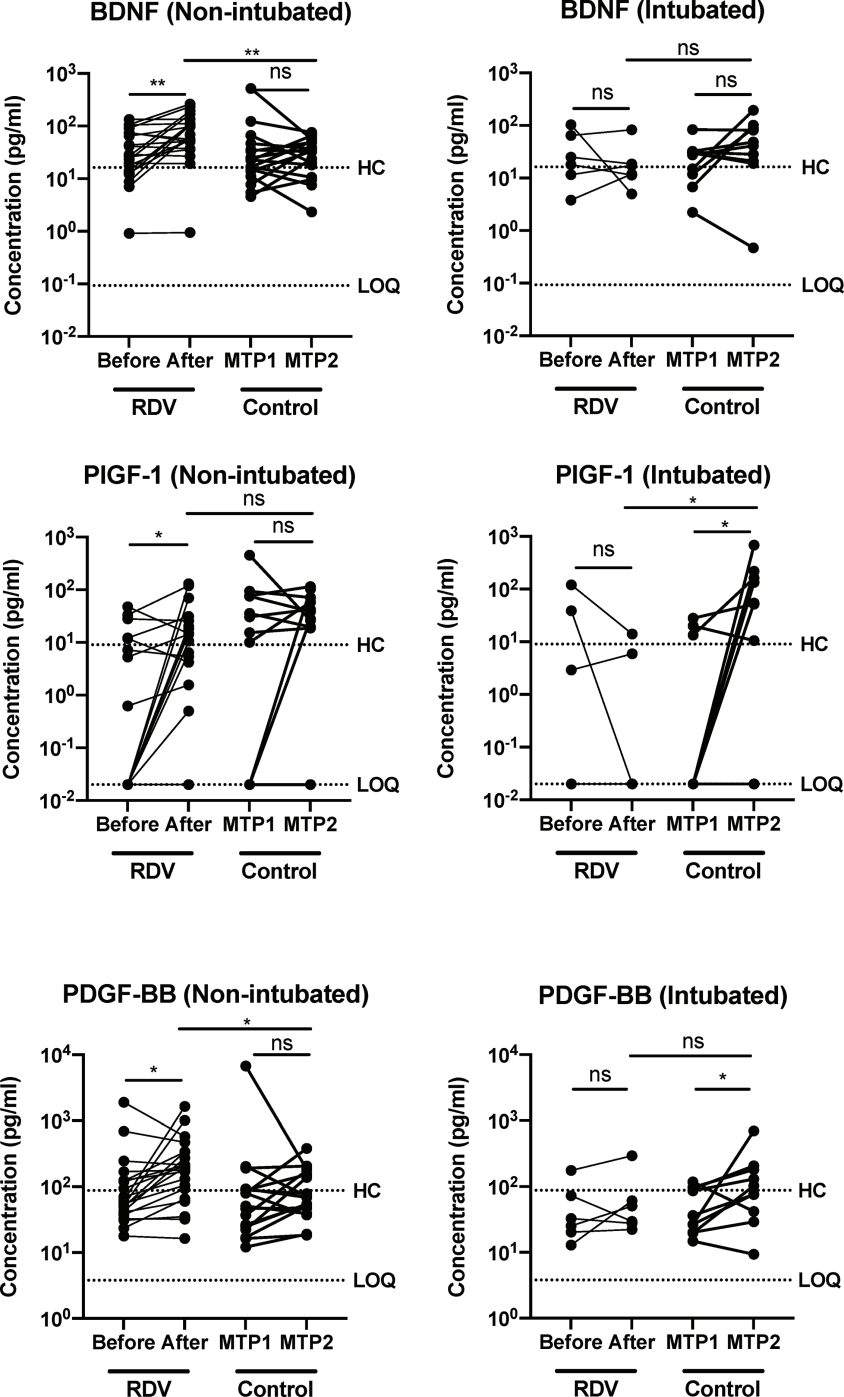
COVID-19 patients responding to remdesivir (RDV) treatment show up-regulation of recovery-associated immune mediators. Growth factors brain-derived neurotropic factor (BDNF), placental growth factor (PIGF-1) and platelet-derived growth factor BB subunit (PDGF-BB) levels in plasma before and after RDV treatment in non-intubated responders (n = 21, median 9 and 16 days post-illness onset (PIO), respectively) and intubated non-responders (n = 6, median 7 and 13 days PIO, respectively), and matched timepoint 1 and 2 in non-RDV-treated non-intubated (n = 17, median 10 and 16 days PIO, respectively) and intubated (n = 10, median 6 and 12 days PIO, respectively) controls. Statistical analyses were performed with Wilcoxon matched-pairs signed rank test when comparing between timepoints, and Mann Whitney U test when comparing across groups (ns, not significant. *P < 0.05; **P < 0.01). Immune mediator levels for healthy controls (n=23) are indicated by the black dotted line. Patient samples with concentration out of measurement range are presented as the value of Limit of Quantification (LOQ).

The effects of RDV in severe COVID-19 patients were first assessed by monitoring the levels of host immune mediators following treatment. We observed that the levels of tissue repair mediators such as BDNF, PDGF-BB and PIGF-1 were significantly increased in responders one week after RDV treatment (**Figure 2**). In contrast, levels of these mediators remain unchanged in the non-responders and the non-intubated controls. In addition, the levels of BDNF, PDGF-BB and PIGF-1 were higher in responders compared to non-responders one-week post treatment (**Supplementary Figure 1**). Interestingly, the levels of PIGF-1 and PDGF-BB were also increased between matched timepoints 1 and 2 in intubated controls (**Figure 2**).

### Responders to Remdesivir Treatment Showed Increase in T_h_2 to T_h_1-Associated Cytokine Ratios

**Figure 3:**
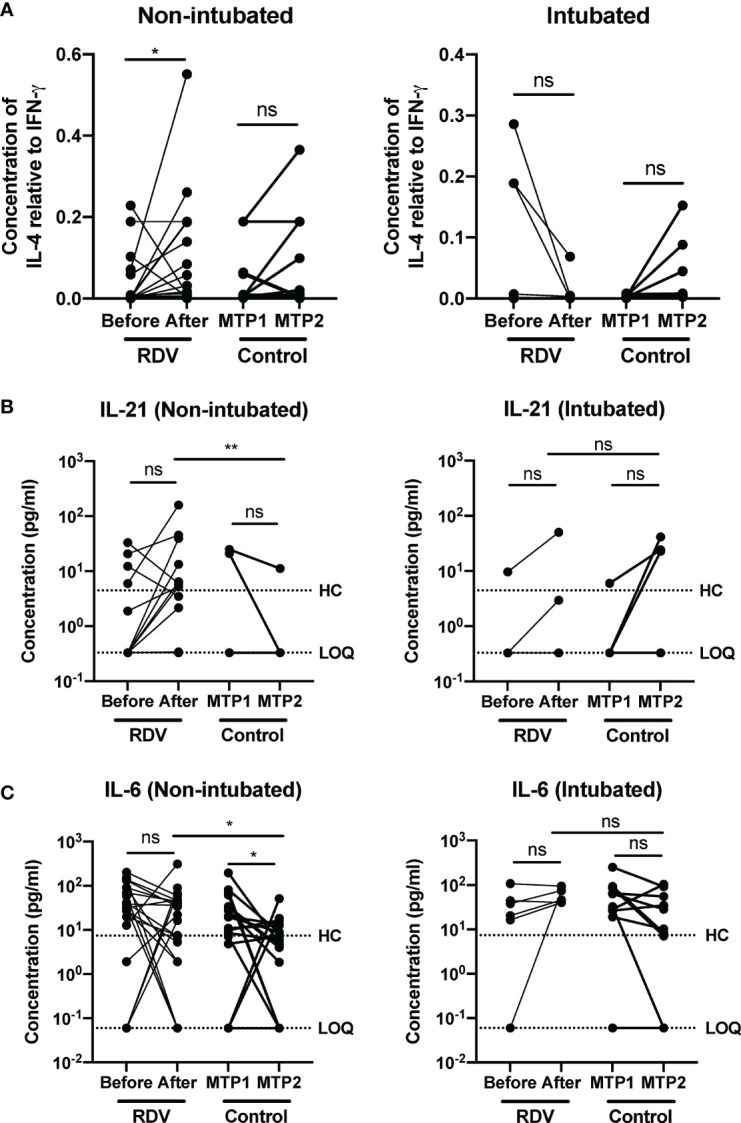
COVID-19 patients responding to remdesivir (RDV) treatment show an increase in T_h_2 to T_h_1 cytokine ratio. Immune mediator levels in inactivated patient plasma was measured with 45-plex Luminex assay. **(A)** IL-4 to IFN-γ ratio of non-intubated responders and intubated non-responders before and one week after treatment with RDV. Statistical analysis was performed with Wilcoxon matched-pairs signed ranked test (ns, not significant; *P < 0.05). **(B)** T_h_2-associated immune mediators IL-21 and **(C)** IL-6 levels in plasma before and after RDV treatment in responders (n = 21, median 9 and 16 days post-illness onset (PIO), respectively) and non-responders (n = 6, median 7 and 13 days PIO, respectively), and matched timepoint 1 and 2 in non-RDV-treated non-intubated (n = 17, median 10 and 16 days PIO, respectively) and intubated (n = 10, median 6 and 12 days post intubation, respectively) controls. Statistical analyses were performed with Wilcoxon matched-pairs signed rank test when comparing between timepoints, and Mann Whitney U test when comparing across groups (ns, not significant; *P < 0.05; **P < 0.01). Immune mediator levels for healthy controls (n=23) are indicated by the black dotted line. Patient samples with concentration out of measurement range are presented as the value of Limit of Quantification (LOQ). ns, not significant.

Multiple reports have highlighted the associations of T cell response with resolution or exacerbation of COVID-19 (11–14). Early induction of IFN-γ producing SARS-CoV-2 specific T helper (T_h_) cells have been associated with milder disease and accelerated viral clearance (15). In contrast, a strong but late T_h_1 response was associated with severe disease (16, 17). To scrutinize if RDV switched the T_h_1/T_h_2 balance in severe patients following treatment, T_h_2/T_h_1 cytokine ratio (defined by IL-4/IFN-γ ratio) was determined and compared between the responders and non-responders. Responders to RDV treatment had an increase in IL-4/IFN-γ ratio, indicating an increase in T_h_2 versus a T_h_1 response, although a T_h_1 response was still predominant. Notably, we also observed 2 responders (9.5%) having a decreasing IL-4/IFN-γ ratio, but this variation could be due to the heterogeneity of our cohort. In contrast, non-RDV-treated controls did not have a similar change in T_h_ cell polarization between matched timepoints 1 and 2 (**Figure 3A**). Importantly, RDV treatment did not affect the IL-4/IFN-γ ratio in non-responders following treatment. Similarly, the difference in the cytokine ratio was not significant in non-RDV-treated patients who progressed to intubation (**Figure 3A**).

To further investigate the effects of RDV on T_h_ cell polarization, the levels of other T_h_1- and T_h_2-associated cytokines were compared in RDV- treated versus control patients. For T_h_2-associated cytokine IL-21, although treatment did not significantly impact IL-21 levels in both treated and control patients, there was an increasing trend for IL-21 in responders (**Figure 3B**). In addition, levels of IL-21 were significantly higher after treatment in responders compared with non-RDV-treated controls at matched timepoint 2. Notably, the levels remained consistent before and after RDV treatment in non-responders (**Figure 3B**). Despite an indication of increasing T_h_2 responses in responders, a decreasing trend approaching healthy baseline was observed for pro-inflammatory IL-6 levels one-week post-treatment (**Figure 3C**). A similar trend was observed in non-intubated controls (**Figure 3C**). Notably, the levels of IL-6 were still significantly higher in responders compared with controls at matched timepoint 2. In contrast, IL-6 levels persisted in non-responders (**Figure 3C**), which corroborated with the sustained levels of pro-inflammatory T_h_1-associated IP-10 (**Supplementary Figure 2**).

**Figure 4:**
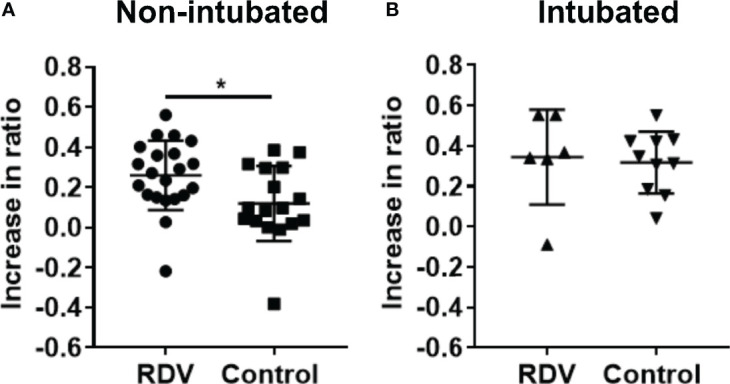
S protein IgG subclasses responses in non-intubated or intubated patients with or without remdesivir (RDV) treatment. **(A)** IgG1, IgG2, IgG3, and IgG4 responses were analysed by screening plasma samples of non-intubated COVID-19 patients with RDV (n = 21) or without RDV (n = 17) treatment, and **(B)** intubated COVID-19 patients with RDV (n = 6) or without RDV (n = 10) treatment at 1:100 dilution against cells expressing the full-length SARS-CoV-2 spike protein, with healthy donors screened in parallel (n = 22). The ratio of combined IgG2 and IgG4 to combined IgG1 and IgG3 response (IgG_2/4_/IgG_1/3_) in RDV-treated patients with or without intubation before and after treatment and also in non-RDV-treated patients at matched time point 1 and 2 were computed and the increase in the ratio from the first to the second time point was plotted. Statistical analyses were performed with Mann Whitney U test (*P < 0.05).

Serology analysis was performed on patient plasma to characterize IgG subclass production and further confirm the increase in T_h_2 responses in the responders. IgG1/3 or IgG2/4 subclasses are associated with T_h_1 or T_h_2 responses, respectively (18–20). Using a flow cytometry-based assay to examine the profile of the IgG subclasses specific against SARS-CoV-2 spike protein (10), an increase in antibody binding capacity was observed across all IgG subclasses in both treated and control patients (**Supplementary Figures 3A–D**). The ratio of IgG2 and IgG4 responses to IgG1 and IgG3 responses (hereinafter IgG_2/4_/IgG_1/3_), indicative of T_h_2/T_h_1 ratios, were increased in both treated and control patients (**Supplementary Figure 3E**). The magnitude of increase in this ratio was significantly higher in RDV-treated responders compared with non-intubated controls (**Figure 4A**), while the fold increase in ratio was similar in non-responders and intubated controls (**Figure 4B**). This result is indicative of an increase in T_h_2 responses during RDV treatment in responders.

### Basal Immune Mediator Levels and RDV Treatment Timing Did Not Influence Disease Progression

One reason for the observed correlation between RDV responsiveness and the levels of the aforementioned immune mediators could be that pre-existing levels of the immune mediators directly affect RDV responsiveness. If so, initial differences in the basal levels of these immune mediators should correlate with RDV responsiveness. However, there were no significant differences in basal levels of immune mediators associated with T cells (IL-2, IL-7, IL-18, IL-1β, IL-21, IL-22, IL-27, IL-4), growth and tissue repair (BDNF, PDGF-BB) or inflammation (IL-6, IP-10 and IL-1RA) before RDV treatment (**Supplementary Figures 4A–C**). Similarly, Receiver Operating Characteristics (ROC) Wilson-Brown analyses on these immune mediators at baseline were also performed, and these prognostic analyses were not significant (**Supplementary Figures 5A–C**). Together, these suggest that the changes in the tissue repair and T_h_ cell polarization responses were induced only after RDV initiation, and that there exist factors which have not yet been measured here that influence both RDV response and cytokine signature.

Since one such factor could be the timing of treatment initiation, we investigated whether the timing of RDV initiation was associated with cytokine responses following treatment. Patients were stratified based on their days PIO during the start of treatment (≤7 [median 6 days PIO] or >7 [median 9 days PIO] days). Other than higher levels of IL-18 in patients treated with RDV at ≤7 days PIO, levels of inflammatory (RANTES, MIP-1β, IL-6, IL-8, IFN-α) and T cell (IFN-γ, IL-1β, IL-12p70, IL-2, IL-5, IL-4, IL-21) -associated cytokines were similar at timepoint 2 (**Supplementary Figures 6A, B**). Thus, the timing of RDV initiation is not the crucial factor involved.

## Discussion

The efficacy of RDV on COVID-19 patients has been investigated in multiple independent studies. In our cohort, the RDV-treated patients were observed to have a longer hospitalization period, which could be a chance occurrence, rather than an effect of RDV. Nevertheless, the other clinical trials with larger sample sizes, like the ACTT-1 (NCT04280705) or SIMPLE-moderate (NCT04292730) trials, showed the clinical benefits of RDV in improving recovery times, without significant mortality benefits (5, 8, 21). However, the subsequent SOLIDARITY trial by World Health Organization (WHO) highlighted the lack of impact of RDV to prevent death in hospitalized COVID-19 patients (22). Here, we showed that the ratio of T_h_2/T_h_1 associated cytokines IL-4 and IFN-γ was increased in favor of IL-4 in responders compared with non-responders. In addition, other T_h_2-associated immune signatures, like increased IL-21 and IL-6 in responders compared with untreated controls indicated a developing type 2 response (23, 24). In addition, serology analysis revealed a significantly higher increase in the ratio of spike protein-specific IgG2/4 antibodies to IgG1/3 antibodies in responders, indicating an upregulation of T_h_2 responses (18). It has been shown that T cells have a prominent role during SARS-CoV-2 infection (12–14, 25). especially during the convalescent phase, where a previous study highlighted the importance of robust memory T cell responses in protection against recurrent episodes of COVID-19 (26). However, it remains debatable if T cell responses are helpful or harmful in COVID-19, or if different T cell subsets function independently to offer protection or mediate pathogenic inflammation during the acute phase of disease. A previous study reported a predominantly T_h_1 response in patients requiring critical care (27), while another study indicated that patients with mild disease had a normal T_h_2 response (28). These results corroborate our findings, where a higher T_h_2 response was observed in responders to RDV treatment. Although the exact function of T_h_2 in severe COVID-19 is unclear, it plays a critical role in reducing inflammation in other lung diseases (29, 30), warranting further studies in COVID-19.

Levels of lung-injury associated tissue repair factors BDNF, PIGF-1 and PDGF-BB were increased only in responders. However, it is unclear if the increase in levels of these mediators was due to reduction in viral load in responders, or indirect effect from the reduction of inflammation due to increase in T_h_2 response. In addition, the non-responders may be experiencing uncontrolled cytokine storm with overactive inflammation that was not reduced by RDV treatment. As such, combinatorial treatment with anti-inflammatory agents such as dexamethasone (31, 32) or baricitinib (33, 34) are likely options to explore when treating severe COVID-19 patients. Indeed, a clinical trial evaluating the efficacy of baricitinib and RDV combinatorial treatment highlighted the superior results compared with RDV alone, with shortening recovery time and improvement in clinical status among COVID-19 patients receiving high-flow oxygen or noninvasive ventilation (33). More importantly, the group receiving baricitinib co-treatment had a reduction in mortality rate by 35% compared to the RDV treatment group over the course of 28 days (33).

In conclusion, responders to RDV treatment were associated with an increase in anti-inflammatory T_h_2 immune response following treatment. It remains to be determined if this effect resulted from reduction of viral load and ensuing inflammation or an indirect immunomodulation by increasing T_h_2 responses. On the other hand, non-responders to RDV treatment was associated with a suboptimal protective T_h_2 response. As SARS-CoV-2 from the *L* clade (SARS-CoV-2 virus reference strain) accounted for 91% of the patients with known virus genotype in this study, the effects of RDV treatment on patients infected with different variants should also be investigated (35). These findings warrant further studies with larger cohorts to define the underlying immune response differences between responders and non-responders of remdesivir that could have prevented or limited the aggravation of COVID-19.

## Data Availability Statement

The original contributions presented in the study are included in the article/**Supplementary Material**. Further inquiries can be directed to the corresponding authors.

## Ethics Statement

The studies involving human participants were reviewed and approved by National Healthcare Group. The patients/participants provided their written informed consent to participate in this study.

## Author Contributions

Y-HC processed, acquired, analyzed and interpreted the data, and wrote the manuscript. BY curated the patients, collected, analyzed and interpreted the data, and wrote the manuscript. S-WF, YSG, and RS-LC, curated the patients, acquired and analyzed the data. YD, S-YT, SK, and PT collected the data. Y-SL, LFPN, DL, and LR conceptualized the study, designed the study protocol, supervised the study and revised the manuscript. All authors contributed to the article and approved the submitted version.

## Funding

This work was supported by Singapore Immunology Network (SIgN) core research grant and the A*STAR COVID-19 Research funding (H/20/04/g1/006) provided to SIgN by the Biomedical Research Council (BMRC). Subject recruitment and sample collection were funded by the National Medical Research Council (NMRC) COVID-19 Research Fund (COVID19RF-001). The SIgN Immunomonitoring Platform is supported by a BMRC IAF 311006 grant and BMRC transition funds #H16/99/b0/011. The SIgN Flow Cytometry and the Multiple Analyte Platforms were supported by a grant from the National Research Foundation, Immunomonitoring Service Platform ISP) (#NRF2017_SISFP09) and the National Research Foundation Singapore (NRF). The funders had no role in the design and conduct of the study; collection, management, analysis and interpretation of the data; preparation, review, or approval of the manuscript; and decision to submit the manuscript for publication. The corresponding author had full access to all the data in the study and takes responsibility for the integrity of the data and the accuracy of the data analysis.

## Conflict of Interest

The authors declare that the research was conducted in the absence of any commercial or financial relationships that could be construed as a potential conflict of interest.
