# Supplementary material for: Differential Cytokine Responses in Hospitalized COVID-19 Patients Limit Efficacy of Remdesivir
Source: Front Immunol. 2021 Jun 28;12:680188. doi: 10.3389/fimmu.2021.680188 (PMC8275132; doi:10.3389/fimmu.2021.680188)
Supplement: Supplementary file 1 [file DataSheet_1.pdf]

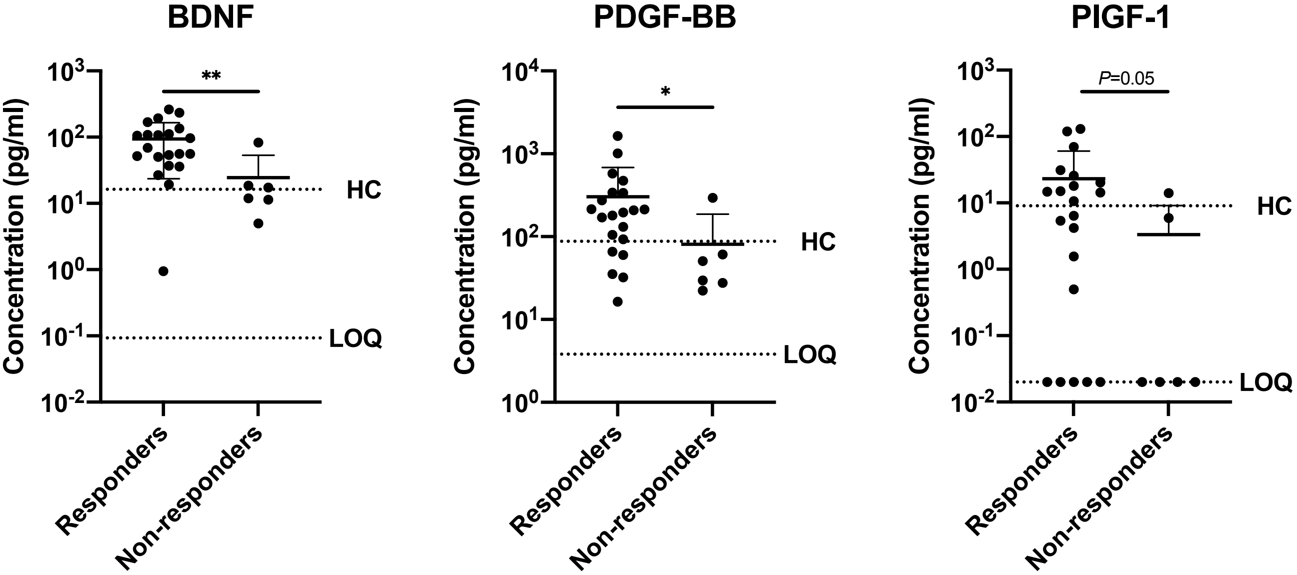

Supplementary Figure 1. Higher recovery-associated immune mediator levels in responders to RDV treatment compared to non-responders. Growth and recovery-associated factors brain-derived neurotropic factor (BDNF), platelet-derived growth factor BB subunit (PDGF-BB) and placental growth factor (PIGF-1) levels in plasma before and one-week after RDV treatment in non-intubated responders (n=21, median 9 and 16 days post-illness onset (PIO), respectively) and intubated non-responders (n=6, median 7 and 13 days PIO, respectively). Statistical analyses were performed with Mann Whitney U test (**P*<0.05; ***P*<0.01). Immune mediator levels for healthy controls (n=23) are indicated by the black dotted line. Patient samples with concentration out of measurement range are presented as the value of Limit of Quantification (LOQ).

Supplementary Figure 1, Chan et al., 2021
