# Supplementary material for: Differential Cytokine Responses in Hospitalized COVID-19 Patients Limit Efficacy of Remdesivir
Source: Front Immunol. 2021 Jun 28;12:680188. doi: 10.3389/fimmu.2021.680188 (PMC8275132; doi:10.3389/fimmu.2021.680188)
Supplement: Supplementary file 2 [file DataSheet_2.pdf]

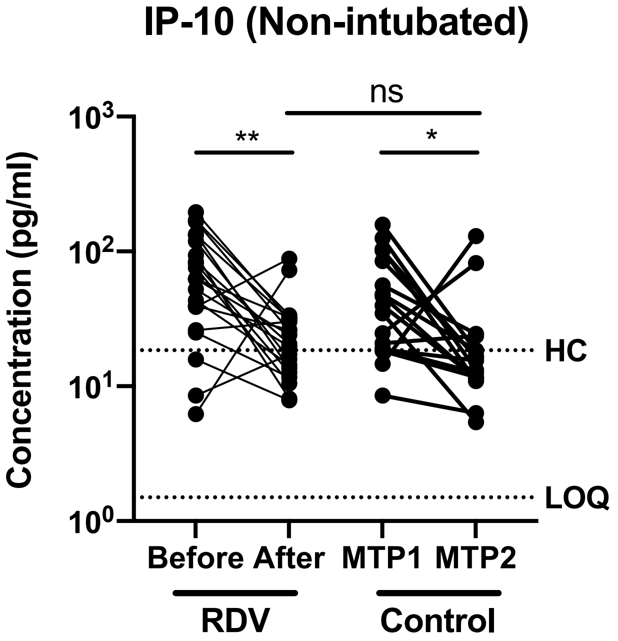

Supplementary Figure 2. Remdesivir treatment results in sustained levels of pro-inflammatory IP-10 in intubated COVID-19 patients. T_h1-associated immune mediator IP-10 levels in plasma before and one-week after RDV treatment in non-intubated responders (n=21, median 9 and 16 days post-illness onset (PIO), respectively) and intubated non-responders (n=6, median 7 and 13 days PIO, respectively), and matched timepoint 1 and 2 in non-RDV-treated non-intubated (n=17, median 10 and 16 days PIO, respectively) and intubated (n=10, median 6 and 12 days PIO, respectively) controls. Statistical analyses were performed with Wilcoxon matched-pairs signed rank test when comparing between timepoints, and Mann Whitney U test when comparing across groups (*P<0.05; **P<0.01). Immune mediator levels for healthy controls (n=23) are indicated by the black dotted line. Patient samples with concentration out of measurement range are presented as the value of Limit of Quantification (LOQ).

Supplementary Figure 2, Chan et al., 2021
