# Supplementary material for: Differential Cytokine Responses in Hospitalized COVID-19 Patients Limit Efficacy of Remdesivir
Source: Front Immunol. 2021 Jun 28;12:680188. doi: 10.3389/fimmu.2021.680188 (PMC8275132; doi:10.3389/fimmu.2021.680188)
Supplement: Supplementary file 3 [file DataSheet_3.pdf]

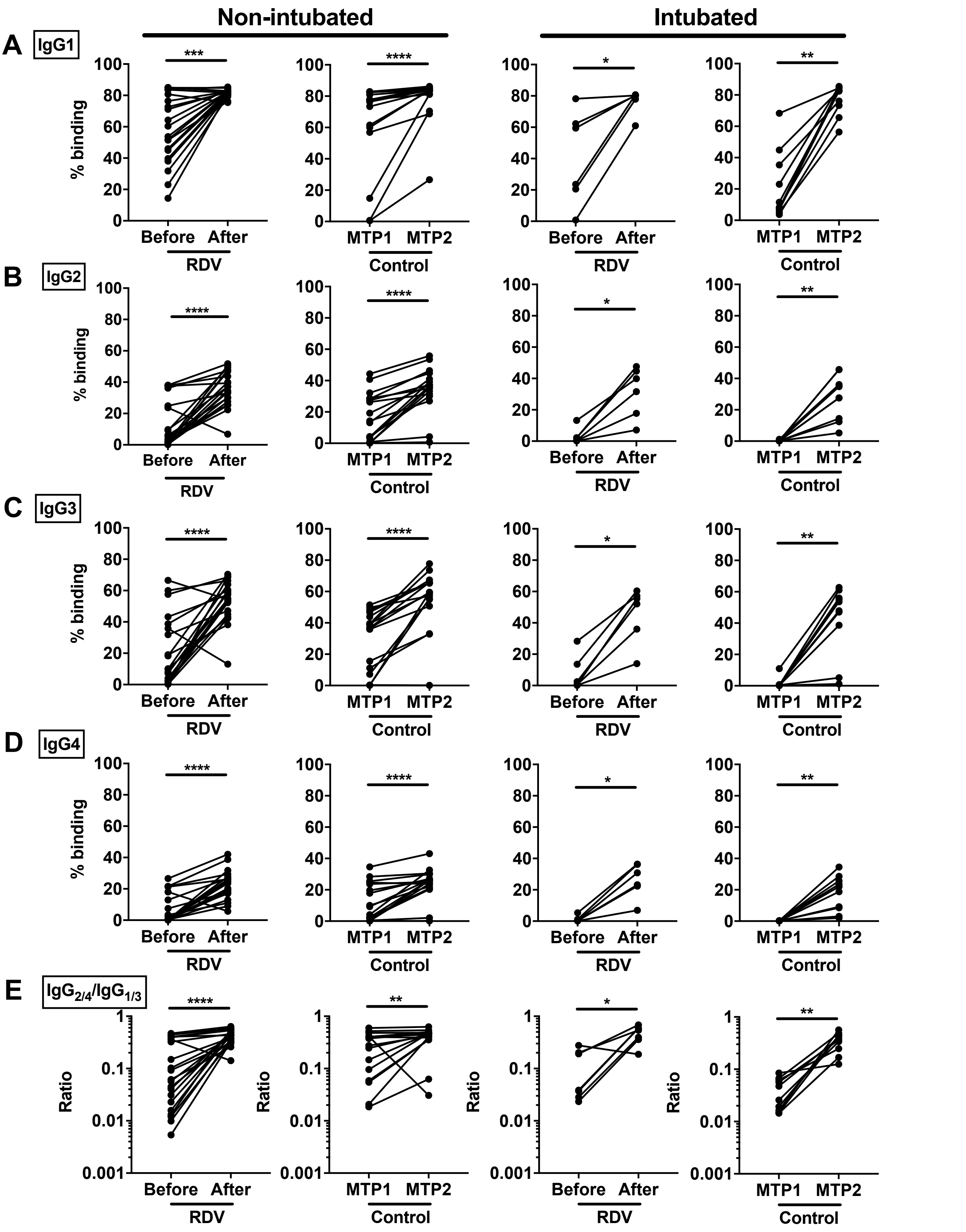


**Supplementary Figure 3. S protein IgG subclasses responses in hypoxia or intubated patients with or without remdesivir (RDV) treatment.** Plasma samples of non-intubated COVID-19 patients with RDV (n=21) or without RDV (n=17) treatment, and intubated COVID-19 patients with RDV (n=6) or without RDV (n=10) treatment were screened at 1:100 dilution against cells expressing the full-length SARS-CoV-2 spike protein, with healthy donors screened in parallel (n=22). **(A)** IgG1, **(B)** IgG2, **(C)** IgG3, and **(D)** IgG4 responses, **(E)** ratio of combined IgG2 and IgG4 to combined IgG1 and IgG3 response ($IgG_{2/4}/IgG_{1/3}$) in RDV-treated patients with or without intubation before and after treatment and also in non-RDV-treated patients at matched time point 1 and 2. Statistical analyses were performed with Wilcoxon matched-pairs signed rank test (*$P<0.05$; **$P<0.01$; ***$P<0.001$; ****$P<0.0001$).

# Supplementary Figure 3, Chan et al., 2021
