# Supplementary material for: Differential Cytokine Responses in Hospitalized COVID-19 Patients Limit Efficacy of Remdesivir
Source: Front Immunol. 2021 Jun 28;12:680188. doi: 10.3389/fimmu.2021.680188 (PMC8275132; doi:10.3389/fimmu.2021.680188)
Supplement: Supplementary file 4 [file DataSheet_4.pdf]

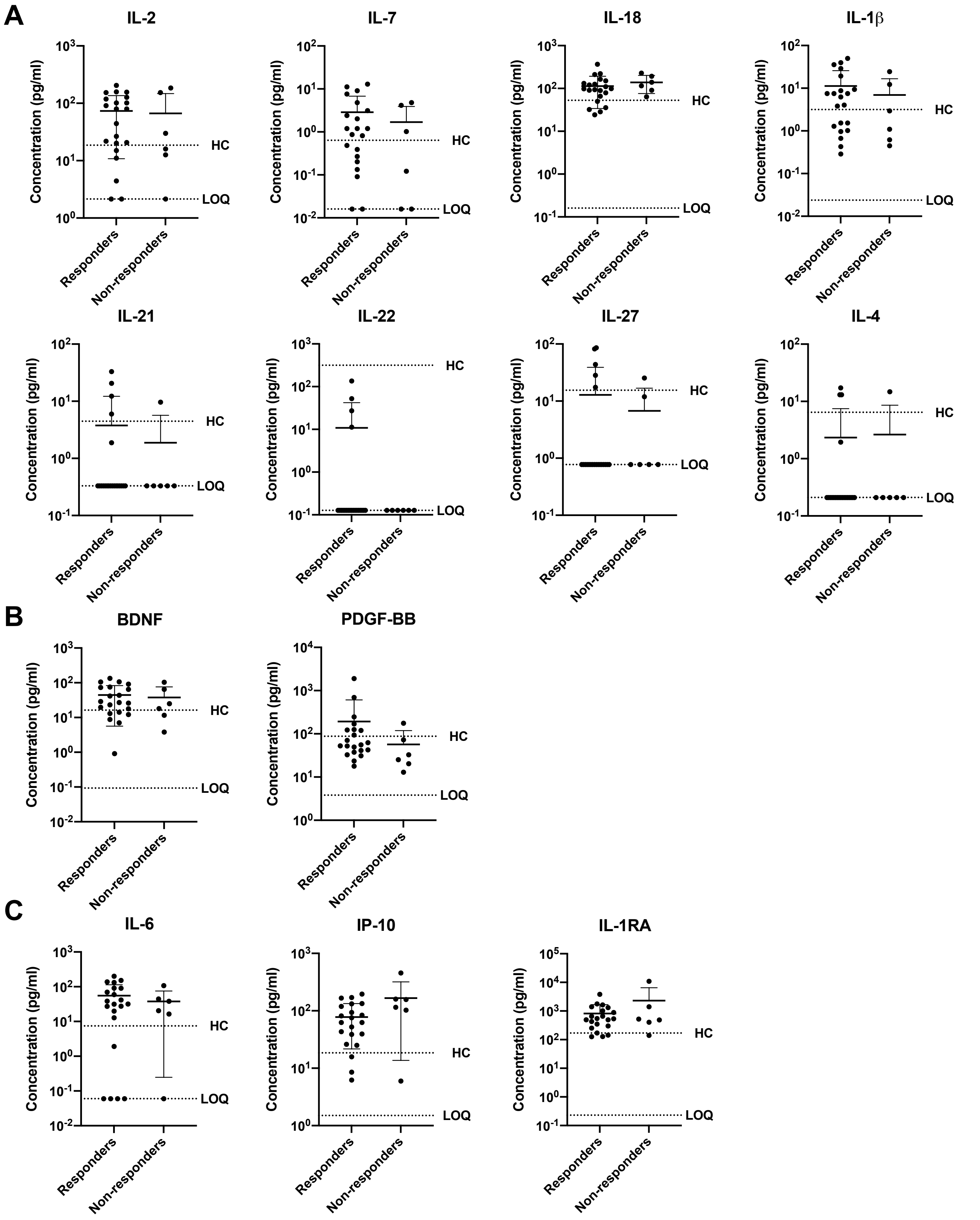

Supplemental Figure 4. Baseline concentrations of immune mediators in COVID-19 patients treated with Remdesivir (RDV). Scatter plots of baseline immune mediator levels in plasma samples of COVID-19 patients before RDV treatment. All patients (n=27) were receiving supplemental oxygen and not intubated prior to treatment. Patients were categorized based on their eventual disease progression to requiring intubation (Non-responders, median 7 days post-illness onset (PIO), n=6), or not (Responders, median 9 days PIO, n=21). Immune mediators were grouped based on their association with **(A)** T cells, **(B)** growth and recovery, or **(C)** inflammation. Statistical analyses were performed with Mann Whitney U test (**P*<0.05; ***P*<0.01). Immune mediator levels for healthy controls (n=23) are indicated by the black dotted line. Patient samples with concentration out of measurement range are presented as the value of Limit of Quantification (LOQ).

Supplementary Figure 4 , Chan et al., 2021
