# Supplementary material for: Differential Cytokine Responses in Hospitalized COVID-19 Patients Limit Efficacy of Remdesivir
Source: Front Immunol. 2021 Jun 28;12:680188. doi: 10.3389/fimmu.2021.680188 (PMC8275132; doi:10.3389/fimmu.2021.680188)
Supplement: Supplementary file 5 [file DataSheet_5.pdf]

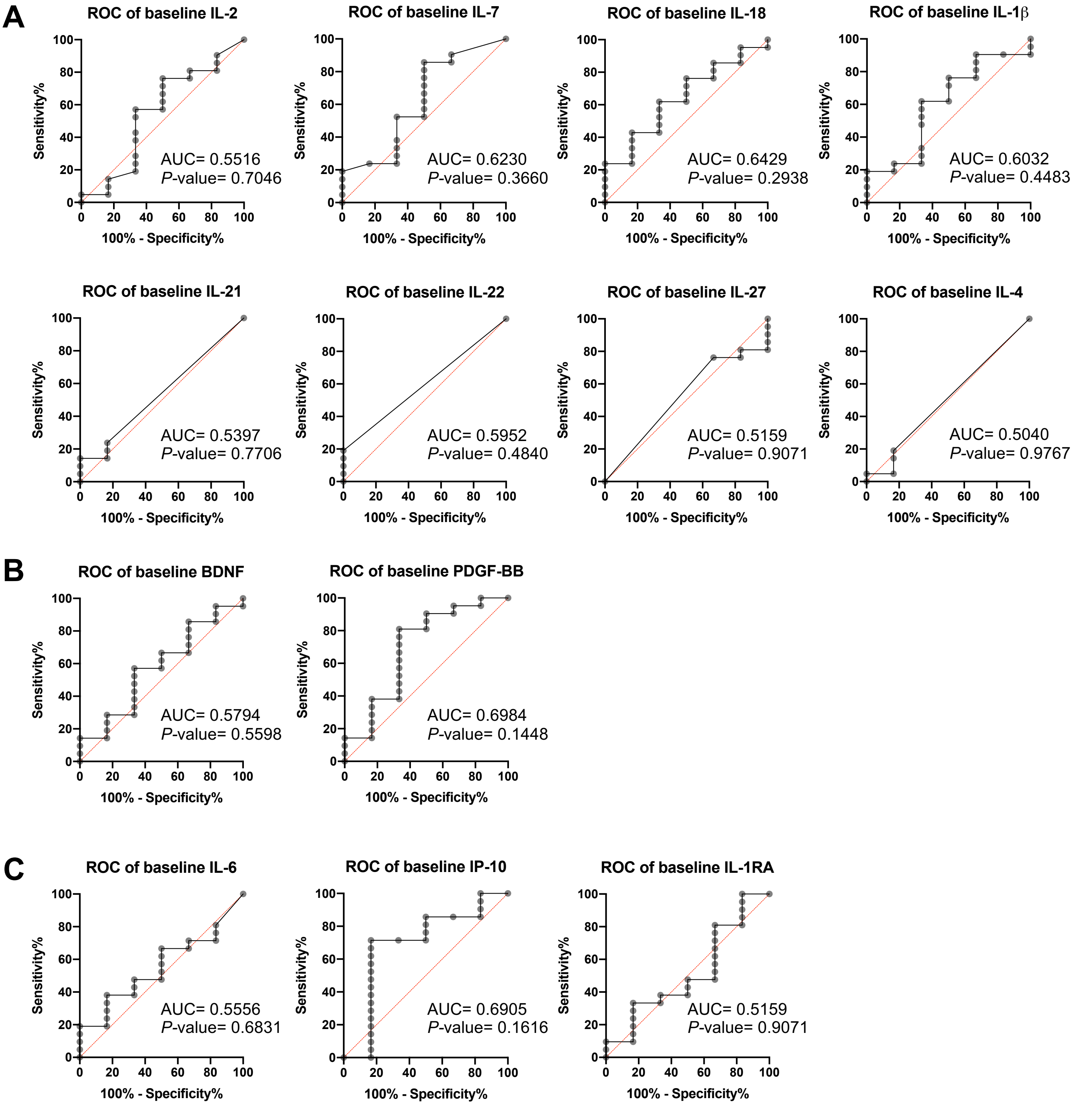

Supplemental Figure 5. Prognostic value of immune mediators at baseline to identify responders from responders from non-responders from patients treated with Remdesivir. Prognostic analyses were performed with Receiver Operating Characteristic (ROC) Wilson-Brown analysis at 95 % Confidence Interval. Baseline levels of **(A)** T-cell associated mediators, **(B)** growth factors and **(C)** inflammatory-associated mediators were measured in plasma samples of COVID-19 patients before RDV treatment. All Patients (n=27) were receiving supplemental oxygen and not intubated prior to treatment. Patients were categorized based on their eventual disease progression to requiring intubation (Non-responders (patients), median 7 days post-illness onset (PIO), n=6), or not (Responders (controls), median 9 days PIO, n=21). Patient samples with concentration out of measurement range are presented as the value of Limit of Quantification (LOQ) for the analysis.

Supplementary Figure 5 , Chan et al., 2021
