# Supplementary material for: Differential Cytokine Responses in Hospitalized COVID-19 Patients Limit Efficacy of Remdesivir
Source: Front Immunol. 2021 Jun 28;12:680188. doi: 10.3389/fimmu.2021.680188 (PMC8275132; doi:10.3389/fimmu.2021.680188)
Supplement: Supplementary file 6 [file DataSheet_6.pdf]

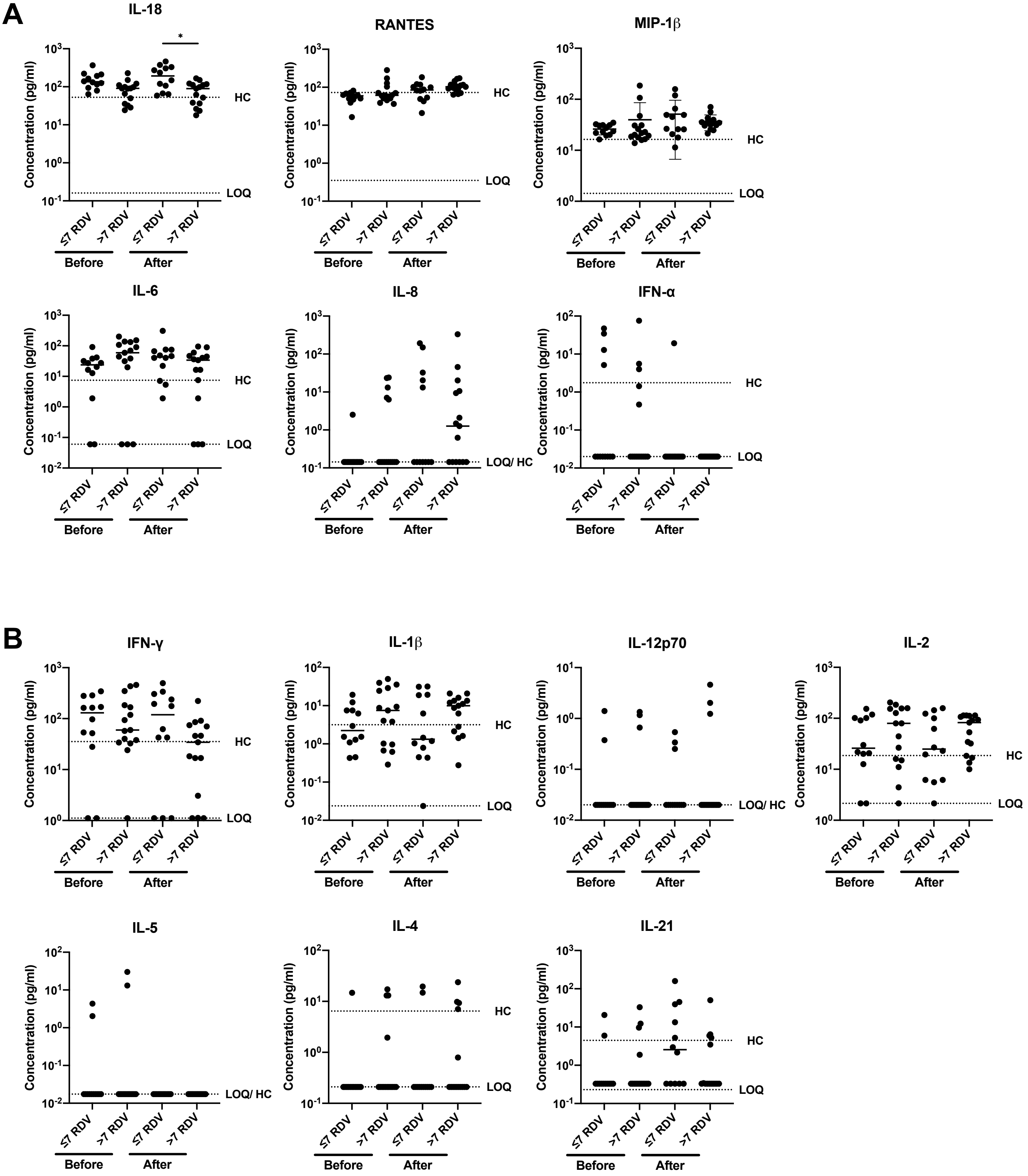

Supplemental Figure 6. Early or late RDV treatment did not impact COVID-19 disease progression. RDV-treated patients were stratified based on their RDV treatment timing relative to days post-illness onset (PIO)(≤7 days (n=12, median 6 days PIO) or >7 days (n=15, median 9 days PIO)). Immune mediator levels in plasma samples collected before and one week after RDV treatment were measured and compared. Immune mediators were grouped based on their associations with **(A)** inflammatory or **(B)** T cell responses. Statistical analyses were performed with Kruskal Wallis test with Dunn's multiple comparison (**P*<0.05). Immune mediator levels for healthy controls (n=23) are indicated by the black dotted line. Patient samples with concentration out of measurement range are presented as the value of Limit of Quantification (LOQ).

Supplementary Figure 6, Chan et al., 2021
