# Supplementary material for: Differential Cytokine Responses in Hospitalized COVID-19 Patients Limit Efficacy of Remdesivir
Source: Front Immunol. 2021 Jun 28;12:680188. doi: 10.3389/fimmu.2021.680188 (PMC8275132; doi:10.3389/fimmu.2021.680188)
Supplement: Supplementary file 7 [file DataSheet_7.pdf]

Supplementary Table 1: Demographics of healthy controls

| Sample ID | Age | Gender |
|---|---|---|
| 4226 | 45 | M |
| 3WLY | 46 | F |
| 4DUY | 45 | M |
| 5UEN | 45 | F |
| 6225 | 57 | M |
| 6233 | 55 | F |
| 1960 | 55 | F |
| 1969 | 56 | F |
| 6280 | 55 | F |
| 1977 | 57 | F |
| 0517 | 67 | F |
| 6209 | 65 | M |
| 1908 | 62 | F |
| 7020 | 72 | M |
| 1899 | 74 | M |
| 1805 | 63 | F |
| 1514 | 70 | M |
| DZQV 2015 | 27 | M |
| 4RQT 2015 | 23 | F |
| 9JD4 2015 | 26 | F |
| CZJE 2015 | 29 | M |
| 5JGU 2015 | 26 | F |
| 4DS3 2016 | 27 | F |
